# University College Hospital

**Published:** 1852-11

**Authors:** 


University College Hospital. *Physicians*—Drs. Walshe, Parkes, and Garrod. *Obstetric Physician*—Dr. Murphy. *Assistant Physicians*—Drs. Jenner and Hare. *Surgeons*—Messrs. Quain and Erichsen. *Consulting Surgeon to Eye Infirmary*—Mr. Quain. *Ophthalmic Surgeon*—Mr. Wharton Jones. *Assistant Surgeons*—Messrs Marshall and Statham.

Faculty of Medicine. *Winter Session.*—Principles and Practice of Medicine, Dr. Walshe. Anatomy and Physiology, Dr. Sharpey. Chemistry, Mr. Graham. Anatomy, Descriptive, Practical, and Surgical, Mr. Ellis. Principles and Practice of Surgery, Mr. Erichsen. Analytical Chemistry, Dr. Williamson. Comparative Anatomy and Zoology, Dr. Grant. Natural Philosophy, Prof. Potter. *Summer Session.*—Botany, Dr. Lindley. Pathological Anatomy, Dr. Jenner. Comparative Anatomy and Zoology, Dr. Grant. Palæo-Zoology, Dr. Grant. Practical Chemistry, Dr. Williamson. Medical Jurisprudence, Dr. Carpenter. Midwifery, &c., Dr. Murphy. Ophthalmic Medicine and Surgery, Mr. Wharton Jones. Materia Medica and Therapeutics, Dr. Garrod.

